# Editorial

**Published:** 1914-08

**Authors:** 


### EDITORIAL

The Business Office of the *Journal-Record* is 23 West Alabama Street.

The Editorial Office is 1014-15 Atlanta National Bank Building.

Address all Business Communications to *Journal-Record of Medicine,* 23 West Alabama Street.

Make remittances payable to *The Journal-Record of Medicine.*

On matters pertaining to the Editorial and Original Communications, address Edgar G. Ballenger, M. D., Atlanta, Ga.

Reprints of Original Articles will be furnished at cost price. Requests for the same should always be made in THE MANUSCRIPT.

We will present, postpaid, on request to each contributor of an original article, twenty (20) marked copies of *The Journal-Record of Medicine* containing such article.

### WHOOPING COUGH.

The Board of Health in Atlanta has taken action in regard to this disease and have requested the physicians to give their counsel in the matter of determining the amount of isolation whooping cough requires.

The important fact is emphasized that whooping cough is a dangerous disease and kills annually more children than measles, scarlet fever and diphtheria combined. This seems to be not generally known and certainly among the laity whooping cough is looked upon as an indifferent disease that children have to suffer with and the sooner they have it and get immunity from it, the better.

The Board gives the information that there is no definite knowledge to be had as to the time when the disease is most infectious nor when the child is not a carrier. As a matter of fact the germ has not been isolated positively and the whole matter of etiology is still in the experimental stage.

Meantime families should be informed of the danger to their own children who suffer from the cough and a quarantine should be maintained until the child is well of the trouble and the cough has disappeared. The whooping has nothing to do with the transmissibility of the infection nor does it indicate in any way the time when the child may be allowed to go with its fellows.

No matter how long a child may be kept from school it should not be allowed to go among other children or be kept indoors as in school so long as it has any cough. There is no reliable treatment for the disease. Outdoor fresh air whenever the child is strong enough to be out is an essential. That and rest make up all that is known as of certain value in this dangerous disease.

The fiction that children should have this and other of the so-called childrens' diseases just because they are children is a most injurious one and should be earnestly combatted wherever it arises. It is as absurd as it would be to say that because many children have to withstand the accident of a broken arm, all children should have their arms broken when they are young. R. R. D.

